# Preliminary Efficacy and Safety of Camrelizumab in Combination With XELOX Plus Bevacizumab or Regorafenib in Patients With Metastatic Colorectal Cancer: A Retrospective Study

**DOI:** 10.3389/fonc.2021.774445

**Published:** 2021-11-25

**Authors:** Hong Zhou, Yuehui Wang, Yanfang Lin, Wenjie Cai, Xiaofeng Li, Xiaomeng He

**Affiliations:** ^1^ Department of Pharmacy, First Hospital of Quanzhou Affiliated to Fujian Medical University, Quanzhou, China; ^2^ Department of Radiology, First Hospital of Quanzhou Affiliated to Fujian Medical University, Quanzhou, China; ^3^ Department of Radiation Oncology, First Hospital of Quanzhou Affiliated to Fujian Medical University, Quanzhou, China; ^4^ Department of Medical Oncology, First Hospital of Quanzhou Affiliated to Fujian Medical University, Quanzhou, China; ^5^ Department of Pharmacy, The First Affiliated Hospital of Dalian Medical University, Dalian, China

**Keywords:** colorectal cancer, camrelizumab, immune checkpoint inhibitor, microsatellite stable, bevacizumab, regorafenib

## Abstract

**Background:**

For a majority of patients with metastatic colorectal cancer (mCRC) with MS stable (MSS) or mismatch repair proficient (pMMR), the role of immunotherapy is undetermined. This study investigated the efficacy and safety of camrelizumab when added to XELOX chemotherapy plus bevacizumab or regorafenib as first-line therapy for mCRC.

**Materials and Methods:**

Medical records of mCRC patients who received camrelizumab and XELOX plus bevacizumab or regorafenib at the First Hospital of Quanzhou Affiliated to Fujian Medical University between June 1, 2019, and April 30, 2021, were retrospectively collected. The objective response rate (ORR), disease control rate (DCR), progression-free survival (PFS), overall survival (OS), and side effects of the drug were recorded and reviewed.

**Results:**

Twenty-five eligible patients received combination therapy, including bevacizumab in 19 patients and regorafenib in 6. Twenty-one patients had pMMR/MSS and one MSI-H. Of the 25 patients who could be evaluated for efficacy, 18 (72%) achieved PR, 6 (24%) achieved SD, and 1 (4%) achieved PD. The ORR and DCR were 72% (18/25) and 96% (24/25), respectively. The median progression-free survival (PFS) was 11.2 months (95% CI 8.9–13.9), and OS had not yet been reached. The combination regimen of regorafenib in six (24%) patients was unassociated with treatment outcomes. Most AEs were either grade 1 or 2, and treatment-related grade 3 toxicities were observed in 8/25 (32%) patients.

**Conclusion:**

Camrelizumab combined with XELOX plus bevacizumab or regorafenib was feasible, producing high rates of responses as first-line therapy in unselected Chinese patients with MSS mCRC. The toxicities were generally tolerable and manageable. Prospective randomized trials with large sample sizes are needed to evaluate these findings.

## Introduction

Immune checkpoint inhibitors (ICIs) have been shown to benefit patients with metastatic colorectal cancer (mCRC) with mismatch repair deficiency (dMMR) or high microsatellite instability (MSI-H) (1, 2). However, PD-1/PDL1 blockade immunotherapy is not effective in pMMR/MSS, which constitutes a large population of patients (3). Ongoing clinical trials are evaluating the efficacy of immunotherapy-based strategies, including chemotherapy, radiotherapy, MEK inhibitors, or other agents in pMMR/MSS mCRC (4). In the REGONIVO study (5), regorafenib combined with nivolumab produced an ORR of 33% (95% CI, 15.6% to 55.3%) in 24 patients with pMMR/MSS refractory mCRC, indicating that anti-angiogenic drugs may enhance the efficacy of immune checkpoint inhibitors. In addition, a single-arm phase II AVETUX trial produced a high ORR of 79.5% in 39 patients, showing the feasibility and early efficacy of avelumab and cetuximab combined with FOLFOX as first-line therapy in RAS/BRAF wildtype MCRC patients (6).

Camrelizumab (SHR-1210) a high-affinity, humanized immunoglobulin and selective IgG4-anti-PD-1 monoclonal antibody, has been approved for the treatment of classical Hodgkin’s lymphoma, advanced hepatic cancer, advanced esophageal cancer, and advanced non-small-cell lung cancer in China (7). Camrelizumab is one of the most widely used anti-PD-1 antibodies for various solid tumors in real-time practice owing to drug accessibility and economic pressure for patients in China. It has shown promising clinical efficacy in several kinds of solid tumors, based on positive efficacy results in clinical trials (8–11), and has also been shown to be effective in MSI-H/dMMR solid tumors (12). The combination of regorafenib and camrelizumab achieved an ORR of 25% in 16 patients with MSS refractory mCRC, indicating the potential benefit of immunotherapy under an appropriately combined therapeutic strategy (13). Hence, we evaluated the efficacy and safety of camrelizumab when added to the first-line XELOX chemotherapy with bevacizumab or regorafenib in patients with metastatic colorectal cancer.

## Materials and Methods

### Patients

The medical records of patients with mCRC who were treated with camrelizumab combined with XELOX plus bevacizumab or regorafenib at the First Hospital of Quanzhou Affiliated to Fujian Medical University between June 1, 2019, and April 30, 2021, were retrieved. The data cutoff date was October 15, 2021. Eligibility for inclusion included histologically-confirmed metastatic colorectal cancer, treated with camrelizumab combined with XELOX plus bevacizumab or regorafenib and no prior systemic therapy, and one or more uni-dimensional measurable lesions according to Response Evaluation Criteria in Solid Tumors (RECIST) version 1.1. Prior adjuvant therapy and radiotherapy or surgery for mCRC were allowed. There were no exclusion criteria. This study was performed in accordance with the Declaration of Helsinki and was approved by the Ethics Review Board of the First Hospital of Quanzhou Affiliated to Fujian Medical University (Fujian Province, China).

### Treatment Methods

Camrelizumab was intravenously administered at a dose of 200 mg on day 1 every 3 weeks. XELOX consisted of oxaliplatin 130 mg/m^2^ on day 1, followed by oral capecitabine 1,000 mg/m^2^ twice daily on days 1 through 14 (28 doses) of a 21-day cycle. Bevacizumab was administered before oxaliplatin at a dose of 7.5 mg/kg on day 1 every 3 weeks. Regorafenib was orally administered 80 mg once per day on day 1 through 21 in a 28-day cycle. Camrelizumab was administered prior to bevacizumab and chemotherapy.

### Efficacy and Toxicities

Tumor responses were evaluated after every two or three cycles of the combination therapy according to the RECIST 1.1 by computed tomography (CT) scan. The objective response rate (ORR) was calculated by pooling the complete response (CR) and partial response (PR) rates. The disease control rate (DCR) was defined as the proportion of patients with a CR, PR, or stable disease (SD). Progression-free survival (PFS) was defined as the time from the beginning of treatment to the first documentation of disease progression, or final follow-up. Overall survival (OS) was defined as the time from the beginning of treatment to the point of death or final follow-up. Toxicities were graded according to the National Cancer Institute Common Toxicity Criteria for Adverse Events, version 5. The data cutoff date was October 15, 2021.

### Statistical Analysis

Statistical analysis was performed using SPSS version 19.0(SPSS, Inc., Chicago, IL, USA). The Kaplan–Meier method was used for PFS and OS. Median follow-up times were computed by the reverse Kaplan-Meier method. Log-Rank test was performed to compare the different groups for PFS univariate analysis. A two-tailed p<0.05 was considered statistically significant in all tests.

## Results

### Patient Characteristics

The baseline characteristics of the 25 patients with mCRC are shown in **Table 1**. The median age was 64 years (range 43-86 years). Among all patients, 13 (52%) were male and 12 (48%) female. Fifteen (60%) were ECOG PS 0 and 8 (32%) ECOG PS 1. In total, 18 (72%) patients were left-sided primary colorectal cancer, and seven (28%) patients right-sided primary colon cancer. Fourteen (56%) patients had Liver metastases. Twenty-one (84%) patients were confirmed as pMMR/MSS. Among the 25 patients, 19 (76%) patients received combination immunotherapy of bevacizumab, and 6 (24%) of regorafenib (**Table 2**).

**Table 1:** Baseline Characteristics.

Characteristics	Patients N (%)
Age (year)	
Median age (range)	64 (43–86)
≥60	15 (60)
<60	10 (40)
Sex	
Male	13 (52)
Female	12 (48)
ECOG PS	
0	15 (60)
1	8 (32)
2	2 (8)
Primary tumor location	
Colon	14 (56)
Right-side	7 (28)
Left-side	7 (28)
Rectum	11 (44)
Type of metastasis	
With liver metastasis	14 (56)
Without liver metastasis	11 (44)
Site of distant metastasis	
Liver	14 (56)
Lung	7 (28)
Lymph nodes	6 (24)
Peritoneum	6 (24)
Peritoneal cavity	8 (32)
Other	5 (20)
MMR or MSI status	
pMMR or MSS	21 (84)
dMMR or MSI-H	1 (4)
Unknown	3 (12)

ECOG PS, Eastern Cooperative Oncology Group performance score.

**Table 2:** Characteristics of individual patients.

No.	Age(year)	Sex	ECOG PS	Cancer type	Sites of metastasis when on treatment	KRAS/BRAF mutation status	MMR or MSI status	Combining regimen	No. of cycles	Response
1	62	F	0	Rectum	Abdomino-pelvic cavity	Wt	pMMR/MSS	Cam+Rego+XELOX	17	PR
2	68	F	1	Rectum	Liver, lung, kidney, peritoneal cavity, RPLN	Wt	pMMR/MSS	Cam+Rego+XELOX	4	PD
3	45	F	1	Right-sided colon	Peritoneal cavity, RPLN, abdominal aortic LN	KRAS Mt	pMMR/MSS	Cam+Rego+XELOX	8	SD
4	70	M	0	Right-sided colon	Liver, lung, peritoneal cavity	KRAS Mt	pMMR/MSS	Cam+Rego+XELOX	15	PR
5	58	M	0	Right-sided colon	Liver	Wt	pMMR/MSS	Cam+Rego+XELOX	14	PR
6	55	M	0	Rectum	Lung	Wt	pMMR/MSS	Cam+Rego+XELOX	15	PR
7	53	F	0	Rectum	Liver	KRAS Mt	pMMR/MSS	Cam+Bev+XELOX	4	PR
8	64	M	0	Right-sided colon	Liver, lung, peritoneal cavity	Unknown	Unknown	Cam+Bev+XELOX	18	PR
9	86	F	2	Right-sided colon	Abdomino-pelvic cavity	Unknown	Unknown	Cam+Bev+XELOX	2	SD
10	78	M	0	Left-sided colon	Liver	Wt	pMMR/MSS	Cam+Bev+XELOX	4	PR
11	65	M	0	Left-sided colon	Liver	Wt	pMMR/MSS	Cam+Bev+XELOX	7	PR
12	64	M	0	Rectum	Lung	Wt	pMMR/MSS	Cam+Bev+XELOX	8	PR
13	69	M	0	Left-sided colon	Peritoneum	KRAS Mt	pMMR/MSS	Cam+Bev+XELOX	6	PR
14	70	M	1	Rectum	Pelvic cavity	Unknown	pMMR/MSS	Cam+Bev+XELOX	10	SD
15	66	F	0	Left-sided colon	Liver	KRAS Mt	dMMR/MSI-H	Cam+Bev+XELOX	14	PR
16	62	F	0	Right-sided colon	Adrenal gland, peritoneal cavity, peritoneum, lymph nodes	Unknown	pMMR/MSS	Cam+Bev+XELOX	6	SD
17	43	F	0	Left-sided colon	Liver	Wt	pMMR/MSS	Cam+Bev+XELOX	8	PR
18	55	F	1	Right-sided colon	Liver, peritoneum, peritoneal cavity	Unknown	pMMR/MSS	Cam+Bev+XELOX	12	PR
19	52	M	0	Rectum	Lung	KRAS Mt	pMMR/MSS	Cam+Bev+XELOX	8	SD
20	57	F	1	Rectum	Liver, peritoneal cavity, bilateral ovarian	KRAS Mt	pMMR/MSS	Cam+Bev+XELOX	11	PR
21	67	M	2	Rectum	Liver, peritoneum	Unknown	Unknown	Cam+Bev+XELOX	5	PR
22	63	M	0	Left-sided colon	Liver, lymph nodes	KRAS Mt	pMMR/MSS	Cam+Bev+XELOX	5	PR
23	54	F	1	Rectum	Liver	Wt	pMMR/MSS	Cam+Bev+XELOX	4	PR
24	77	F	1	Rectum	Lung, bone, cervical and pelvic wall	Wt	pMMR/MSS	Cam+Bev+XELOX	3	PR
25	76	M	1	Left-sided colon	Abdominal wall, abdomino-pelvic cavity	Wt	pMMR/MSS	Cam+Bev+XELOX	8	SD

ECOG PS, Eastern Cooperative Oncology Group performance status; F, female; M, male; Mt, mutant; Wt, wild-type; Cam, camrelizumab; Bev, bevacizumab; Rego, regorafenib; XELOX, capecitabine and oxaliplatin; PR, partial response; SD, stable disease; PD, progression of disease; RPLN, retroperitoneal lymph node.

### Efficacy

**Figure 1:**
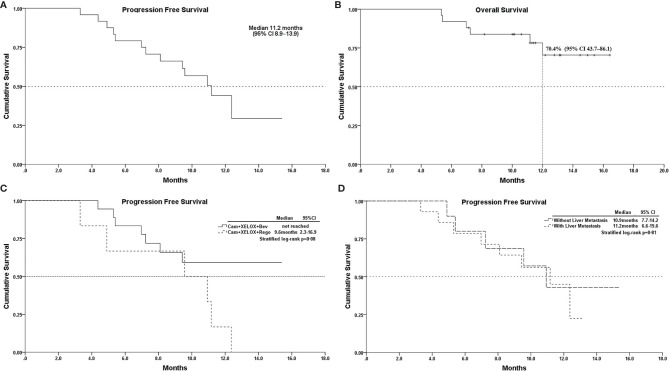
**(A)**, Kaplan–Meier survival curves of progression-free survival; **(B)**, overall survival; **(C)**, PFS of Cam+XELOX+Bev *vs* Cam+XELOX+Rego (p > 0.05); and **(D)**, PFS in patients with or without liver metastasis (p > 0.05); Cam, camrelizumab; Bev, bevacizumab; Rego, regorafenib; XELOX, capecitabine and oxaliplatin.

Of the 25 patients, none achieved CR; 18(72%) experienced partial responses and 6(24%) experienced stable disease as best responses, while one (4%) patient had progressive disease. The ORR and DCR were 72% (18/25) and 96% (24/25), respectively (**Table 3**). The median follow-up for overall survival was 11.5months (95% CI10.3–12.7). The median progression-free survival(mPFS) was 11.2 months (95% CI 8.9–13.9) (**Figure 1A**). The OS was still immature and one-year OS rates were 70.4% (95% CI 43.7–86.1) (**Figure 1B**). In addition, the mPFS of the regorafenib-containing regimen was 9.6 months and PFS of bevacizumab-containing regimen has not yet been reached, although the difference was considered not statistically significant (p = 0.08, **Figure 1C**). The difference in patients with liver metastasis did not reach statistical significance compared with patients without liver metastasis (11.2 *vs.* 10.9months, p = 0.81, **Figure 1D**).

**Table 3:** Tumor response.

Response	Patients N (%)
CR	0
PR	18 (72)
SD	6 (24)
PD	1 (4)
ORR	18 (72)
DCR	24 (96)

CR, complete response; PR, partial response; SD-stable disease; PD, progression of disease; ORR, objective response rate; DCR, disease control rate.

### Safety

All 25 patients were assessed for toxicities. The overall incidence of any grade toxicity was 72% (18/25). Common treatment-related adverse events (AEs) of any grade were neutropenia (36%) reactive cutaneous capillary endothelial proliferation (32%), decreased platelet count (28%), hand-foot syndrome (28%), and liver dysfunction (16%). Grade ≥3 treatment-related AEs occurred in 8(32%) patients. They included neutropenia, gastric hemorrhage, hand-foot syndrome, hyperglycemia, and elevated ALT. A fatal event of gastrointestinal perforation complicated by febrile neutropenia occurred in a patient treated with regorafenib. Treatment was switched from capecitabine to raltitrexed due to intolerable grade 3 hand-foot syndrome in three (12%) patients. The most common camrelizumab-related adverse events were reactive cutaneous capillary endothelial proliferation (32%) and thyroid dysfunction (24.0%), and all of them were either grade 1 or 2. A new camrelizumab-related adverse event, mild bilateral optic disc disease, was observed. Details of the adverse events are presented in **Table 4**.

**Table 4:** Adverse events.

Adverse event	Grade 1-2, N (%)	Grade ≥3, N (%)	Any grade, N (%)
Neutropenia	8 (32)	1 (4)	9 (36)
Decreased platelet count	6 (24)	1 (4)	7 (28)
Nausea and Vomiting	1 (4)	1 (4)	2 (8)
Liver dysfunction	2 (8)	2 (8)	4 (16)
Hand-foot syndrome	4 (16)	3 (12)	7 (28)
Gastric hemorrhage	0	1 (4)	1 (4)
Diarrhea	1 (4)	1 (4)	2 (8)
Fever	2 (8)	1 (4)	3 (12)
Hyperthyroidism	4 (16)	0	4 (16)
Hypothyroidism	2 (8)	0	2 (8)
Hyperglycemia	1 (4)	1 (4)	2 (8)
RCCEP	8 (32)	0	8 (32)
Vision changes	1 (4)	0	1 (4)
Myocarditis	2 (8)	0	2 (8)
Infusion-related reactions	2 (8)	0	2 (8)
ALL	16 (64)	8 (32)	18 (72)

RCCEP, reactive cutaneous capillary endothelial proliferation.

## Discussion

Although recently, rapid development has been made in the field of immunotherapy (14, 15), the first-line standard treatment for metastatic colorectal cancer (mCRC) is 5FU-based chemotherapies, with or without anti-angiogenic agents. ICI monotherapy is efficacious in the treatment of mCRC with dMMR/MSI-H. The role of the combination of PD-1 blockade with VEGF inhibition has been investigated in MSS mCRC. Adding atezolizumab to FP/BEV (standard of care) as first-line maintenance treatment for patients with BRAF wild-type mCRC did not lead to improvement in the outcomes for efficacy (16). However, in the AVETUX trial, avelumab and cetuximab in combination with FOLFOX in patients with previously untreated mCRC produced a high response rate of 79.5%, disease control rate of 92.3% and mPFS of 11.1months in 39 patients with RAS/BRAF wild-type MSS mCRC (6). In this study, a high ORR of 72% and a DCR of 96% were recorded. The mPFS of 11.2 months is as long as that of the AVETUX trial (11.1 months), which is much better than that of bevacizumab plus XELOX chemotherapy (9.4 months) as first-line therapy in patients with mCRC (17). The main factors leading to different efficacies may be that populations in the AVETUX trial included more patients with left-sided tumors (91%) compared to our study (68.8%), and all patients were RAS/BRAF wild-type. Patients with RAS with left-sided mCRC had significantly superior PFS, OS, and ORR compared with patients with right-sided tumors (18), and cetuximab plus FOLFIRI versus bevacizumab as first-line treatment clearly benefitted patients with left-sided tumors (18, 19).

Several studies have suggested that liver metastases are predictive of a lack of benefit from PD-1/PD-L1 inhibitors in MSS mCRC (5, 20). The liver is considered an immunologically-tolerant organ that is characterized by a much higher proportion of immunosuppressive cells (21). In the present study, the difference in patients with liver metastasis was considered not statistically significant compared with those without liver metastasis, probably due to the small sample size.

Recently, in the REGONIVO trial, the combination of regorafenib and nivolumab achieved a robust response rate of 33% in pMMR/MSS refractory mCRC (5). More and more research focused on the regorafenib plus ICIs in MSS refractory mCRC, the current conclusion is controversial due to the small sample size and inconsistency (5, 13, 22–24). In this study, we observed that the mPFS of regorafenib-containing therapies was not as long as that of bevacizumab-containing therapies. Moreover, capecitabine-related hand-foot syndrome (HFS), one of the causes of the switch in three treatments, was in a regorafenib-containing regimen. HFS is a common skin reaction to capecitabine with rates of any grade, of 22%–77% (25). Similarly, regorafenib-associated hand-foot skin reactions occurred at a rate of 61% overall and 20% at grade 3 (26, 27). Regorafenib combined with capecitabine treatment should be used cautiously due to the risk of overlapping skin toxicity. Therefore, this might suggest that as first-line therapy for mCRC, regorafenib may not be a suitable choice for combination with XELOX chemotherapy in future trials.

The combination strategy indicated a potential benefit in terms of ORR and PFS with a acceptable safety profile. Despite its strengths, there are some limitations to consider. First, this is a retrospective pilot study with a small sample size, reflecting its preliminary nature. Second, although antitumor response was observed in mCRC in spite of RAS mutations, a small number of patients had unknown RAS or BRAF status before the beginning of the combination treatment. Besides, the MMR or MSI status of three patients was unknown. Finally, even if the same chemotherapy regimen was adopted, different courses of treatment and varying follow-up intervals may have increased the heterogeneity. Thus, the findings need to be further assessed in a large prospective study.

In conclusion, our study differs from previous immunotherapy study in MSS mCRC based on the study populations and the novel combination regimen. In present study showed the addition of camrelizumab to the first-line XELOX chemotherapy with bevacizumab have demonstrated high response rates, and this immunotherapy combination was practical and helpful in unselected patients with mCRC. Further randomized trials with large sample sizes for this combination strategy are warranted.

## Data Availability Statement

The raw data supporting the conclusions of this article will be made available by the authors, without undue reservation.

## Ethics Statement

This study was approved by the Ethics Review Board of the First Hospital of Quanzhou Affiliated to Fujian Medical University, East Road 250, Quanzhou 362000, Fujian Province, China. Written informed consent for participation was not required for this study, in accordance with the national legislation and institutional requirements.

## Author Contributions

WC, XL, and XH designed the project. HZ, YL, and XH collected patients’ information and wrote the manuscript. WC and XL provided study material or patient. YW evaluated tumor responses. All authors contributed to the article and approved the submitted version.

## Conflict of Interest

The authors declare that the research was conducted in the absence of any commercial or financial relationships that could be construed as a potential conflict of interest.

## Publisher’s Note

All claims expressed in this article are solely those of the authors and do not necessarily represent those of their affiliated organizations, or those of the publisher, the editors and the reviewers. Any product that may be evaluated in this article, or claim that may be made by its manufacturer, is not guaranteed or endorsed by the publisher.
