# Supplementary figures and images for: Preliminary Efficacy and Safety of Camrelizumab in Combination With XELOX Plus Bevacizumab or Regorafenib in Patients With Metastatic Colorectal Cancer: A Retrospective Study
Source: Front Oncol. 2021 Nov 25;11:774445. doi: 10.3389/fonc.2021.774445 (PMC8657606; doi:10.3389/fonc.2021.774445)

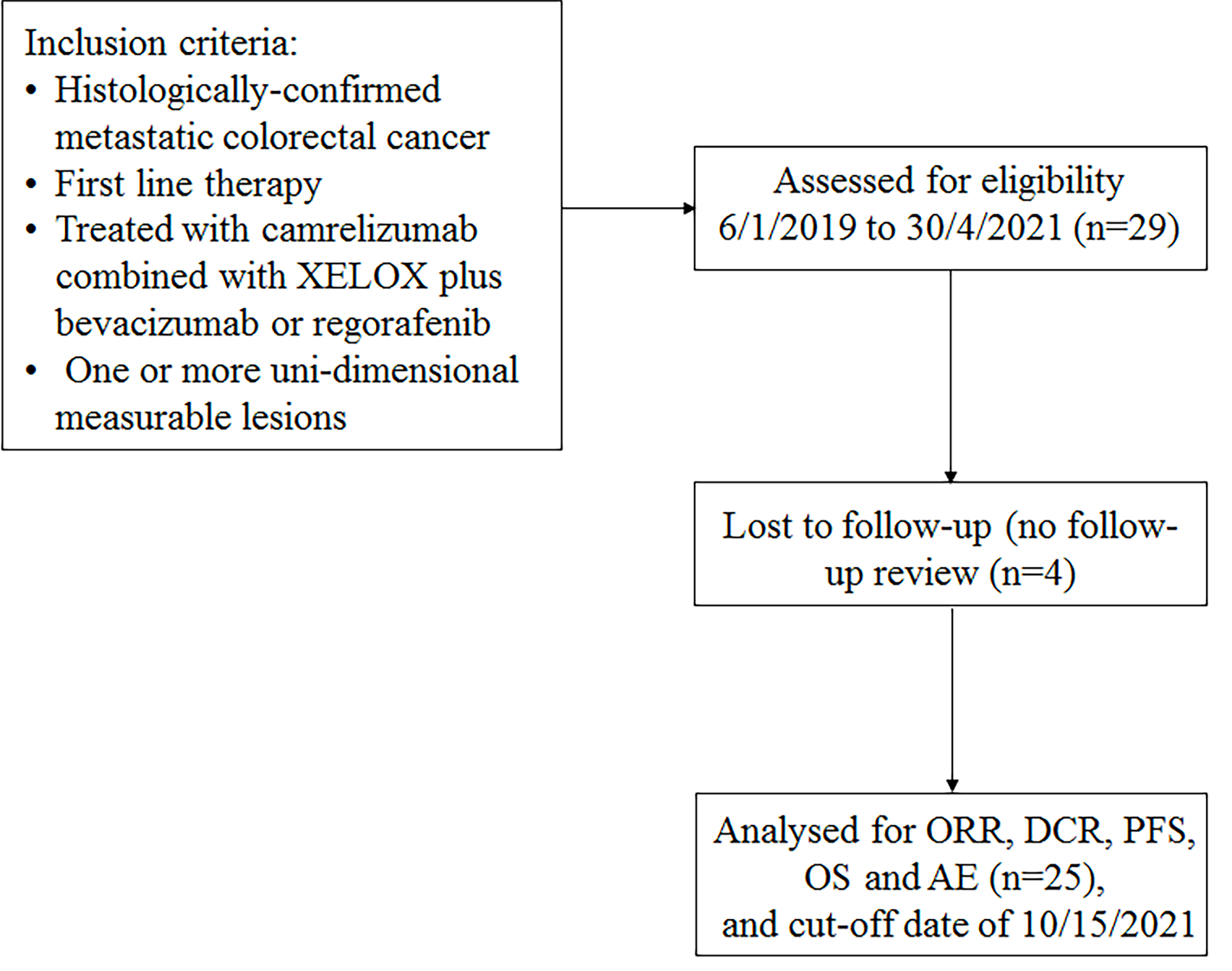

Supplement: Supplementary file 1 [file Image_1.jpeg]
